# Human T-Lymphotropic Virus (HTLV): Epidemiology, Genetic, Pathogenesis, and Future Challenges

**DOI:** 10.3390/v17050664

**Published:** 2025-05-01

**Authors:** Francesco Branda, Chiara Romano, Grazia Pavia, Viola Bilotta, Chiara Locci, Ilenia Azzena, Ilaria Deplano, Noemi Pascale, Maria Perra, Marta Giovanetti, Alessandra Ciccozzi, Andrea De Vito, Angela Quirino, Nadia Marascio, Giovanni Matera, Giordano Madeddu, Marco Casu, Daria Sanna, Giancarlo Ceccarelli, Massimo Ciccozzi, Fabio Scarpa

**Affiliations:** 1 Unit of Medical Statistics and Molecular Epidemiology, Università Campus Bio-Medico di Roma, 00128 Rome, Italy; chiara.romano@unicampus.it (C.R.); violabilotta99@gmail.com (V.B.); m.ciccozzi@unicampus.it (M.C.); 2 Unit of Clinical Microbiology, Department of Health Sciences, “Magna Græcia” University of Catanzaro—“Renato Dulbecco” Teaching Hospital, 88100 Catanzaro, Italy; graziapavia@unicz.it (G.P.); quirino@unicz.it (A.Q.); nmarascio@unicz.it (N.M.); mmatera@unicz.it (G.M.); 3 Department of Biomedical Sciences, University of Sassari, 07100 Sassari, Italy; c.locci3@phd.uniss.it (C.L.); i.deplano@phd.uniss.it (I.D.); npascale@uniss.it (N.P.); m.perra9@studenti.uniss.it (M.P.); darsanna@uniss.it (D.S.); 4 Department of Veterinary Medicine, University of Sassari, 07100 Sassari, Italy; iazzena@uniss.it (I.A.); marcasu@uniss.it (M.C.); 5 Department of Chemical, Physical, Mathematical and Natural Sciences, University of Sassari, 07100 Sassari, Italy; 6 Department of Science and Technologies for Sustainable Development and One Health, Università Campus Bio-Medico di Roma, 00128 Rome, Italy; giovanetti.marta@gmail.com; 7 Instituto Rene Rachou, Fundação Oswaldo Cruz-FIOCRUZ, Belo Horizonte 30190-009, MG, Brazil; 8 Facoltà Dipartimentale di Scienze e Tecnologie per lo Sviluppo Sostenibile e One Health, Università Campus Bio-Medico di Roma, 00128 Rome, Italy; alessandra.ciccozzi@unicampus.it; 9 Unit of Infectious Diseases, Department of Medicine, Surgery and Pharmacy, University of Sassari, 07100 Sassari, Italy; andreadevitoaho@gmail.com (A.D.V.); giordano@uniss.it (G.M.); 10 Department of Public Health and Infectious Diseases, University of Rome Sapienza, 00161 Rome, Italy; giancarlo.ceccarelli@uniroma1.it; 11 Azienda Ospedaliero Universitaria Umberto I, 00185 Rome, Italy

**Keywords:** HTLV, deltaretrovirus, adult T-cell leukemia/lymphoma, HAM/TSP, epidemiology, molecular diagnostics, genomics, genetic characterization

## Abstract

Human T-lymphotropic viruses (HTLVs) are deltaretroviruses infecting millions of individuals worldwide, with HTLV-1 and HTLV-2 being the most widespread and clinically relevant types. HTLV-1 is associated with severe diseases such as adult T-cell leukemia/lymphoma (ATL) and HTLV-1-associated myelopathy/tropical spastic paraparesis (HAM/TSP), while HTLV-2 shows a lower pathogenic potential, with occasional links to neurological disorders. HTLV-3 and HTLV-4, identified in Central Africa, remain poorly characterized but are genetically close to their simian counterparts, indicating recent zoonotic transmission events. HTLVs replicate through a complex cycle involving cell-to-cell transmission and clonal expansion of infected lymphocytes. Viral persistence is mediated by regulatory and accessory proteins, notably Tax and HBZ in HTLV-1, which alter host cell signaling, immune responses, and genomic stability. Integration of proviral DNA into transcriptionally active regions of the host genome may contribute to oncogenesis and long-term viral latency. Differences in viral protein function and intracellular localization contribute to the distinct pathogenesis observed between HTLV-1 and HTLV-2. Geographically, HTLV-1 shows endemic clusters in southwestern Japan, sub-Saharan Africa, the Caribbean, South America, and parts of the Middle East and Oceania. HTLV-2 is concentrated among Indigenous populations in the Americas and people who inject drugs in Europe and North America. Transmission occurs primarily via breastfeeding, sexual contact, contaminated blood products, and, in some regions, zoonotic spillover. Diagnostic approaches include serological screening (ELISA, Western blot, LIA) and molecular assays (PCR, qPCR), with novel biosensor and AI-based methods under development. Despite advances in understanding viral biology, therapeutic options remain limited, and preventive strategies focus on transmission control. The long latency period, lack of effective treatments, and global neglect complicate public health responses, underscoring the need for increased awareness, research investment, and targeted interventions.

## 1. Introduction

The Human T-lymphotropic viruses (HTLVs) are a group of retroviruses classified within the *Deltaretrovirus* genus of the *Retroviridae* family. Currently, four primary subtypes have been identified: HTLV-1, HTLV-2, HTLV-3, and HTLV-4 [1,2,3,4]. HTLV-1 was the first human retrovirus to be isolated, identified in the early 1980s from T-cell cultures derived from patients with adult T-cell leukemia (ATL) and cutaneous T-cell lymphoma [1,2]. Globally, it is estimated that 10–20 million individuals are HTLV-1 infected, with endemic clusters observed in regions such as southwestern Japan, sub-Saharan Africa, South America, the Caribbean, the Middle East, and parts of Australo-Melanesia [5,6,7,8]. Transmission patterns differ across geographic regions; in Japan, mother-to-child transmission via breastfeeding is the primary mode of spread, whereas in South America and Africa, horizontal transmission, including sexual contact and blood exposure, plays a more significant role [9,10,11]. Although the mechanisms underlying HTLV-1 pathogenesis remain incompletely understood, the virus is known to target multiple immune cell populations, including dendritic cells, macrophages, monocytes, CD8+ T lymphocytes, and predominantly CD4+ T lymphocytes [12]. Following primary infection, which occurs exclusively through cell-to-cell transmission [13], viral persistence is primarily maintained by clonal expansion of infected CD4+ T cells rather than by de novo infection [14]. While the majority of HTLV-1-infected individuals remain asymptomatic, approximately 2–10% develop severe conditions, including ATL, HTLV-1-Associated Myelopathy/Tropical Spastic Paraparesis (HAM/TSP), and HTLV-1-associated uveitis (HU), largely due to dysregulated immune responses [12]. HTLV-2, although distinct in its pathogenic profile from HTLV-1 [15], has been linked to an increased risk of chronic neurological disorders [16]. First isolated in 1982 from patients with hairy cell leukemia [14], HTLV-2 primarily circulates among indigenous populations in the Americas and injection drug users in North America and Europe [17]. Over the years, numerous studies have explored the differences between the two subtypes. Their regulatory proteins, such as Tax and their antisense-encoded factors, the basic leucine zipper factor (HBZ) for HTLV-1 and APH-2 for HTLV-2, engage distinct cellular pathways and signaling mechanisms which are believed to underlie the differences in their pathogenic potential [18,19]. More recently, HTLV-3 and HTLV-4 have been identified in individuals who hunt nonhuman primates in Cameroon [3,20]. While HTLV-3 shares some functional similarities with HTLV-1, particularly in the Tax protein [21], no associated diseases have been documented in individuals infected with either HTLV-3 or HTLV-4 [22,23]. However, given their genetic similarity to HTLV-1 and HTLV-2 [24], ongoing research is warranted to assess their potential clinical and public health implications.

Although HTLV-1 and HTLV-2 infections are recognized as a major global health concern, particularly in low-socioeconomic and low-resource regions [6,7], research efforts toward the development of virus-specific vaccines and antiretroviral therapies remain limited. At present, medical interventions for infected individuals primarily focus on managing HTLV-related diseases rather than targeting the infection itself. Preventive measures vary by region and emphasize reducing transmission risk through public health initiatives [25]. These include advising positive mothers to avoid breastfeeding [26], promoting condom use to minimize sexual transmission [27], and advocating for the use of leukoreduced blood products in transfusions [28], which have demonstrated a 93% reduction in transmission risk. The absence of effective antiviral therapies and vaccines, combined with the long latency period of HTLV-associated diseases, poses significant challenges for disease control and prevention. Diagnostic tools remain limited in many high-burden regions, further complicating public health efforts [29].

The objective of this review is to provide a comprehensive analysis of HTLVs, with a particular focus on their epidemiology, pathogenesis, and the challenges they present for public health and clinical management. We aim to discuss the recent advancements in the understanding of HTLVs biology and disease mechanisms while highlighting gaps in the current knowledge. This review will also explore future research priorities and potential strategies for improving the diagnosis, treatment, and prevention of HTLV-associated diseases. Given the increasing recognition of HTLVs as neglected tropical pathogens, it is crucial to stimulate further research and promote global awareness of this important public health issue.

## 2. Virological Characteristics of HTLVs and Infectious Cycle

**Figure 1:**
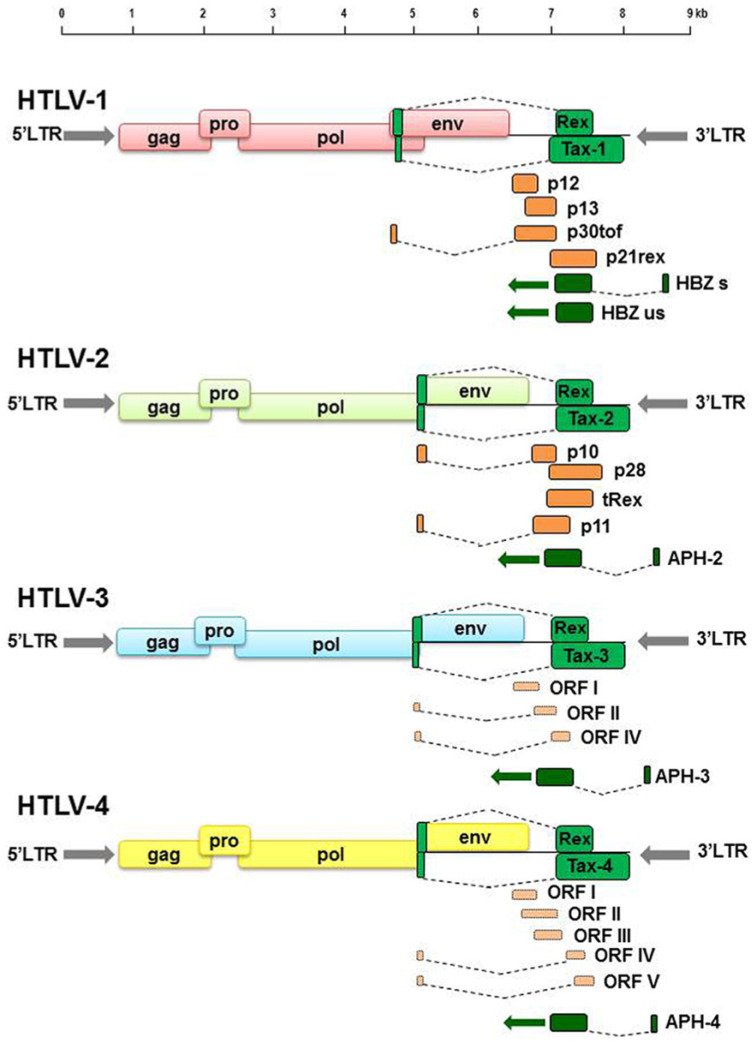
Diagram of the genomic organization of HTLV-1, HTLV-2, HTLV-3, and HTLV-4. Green boxes represent ORFs coding for regulatory proteins, dark orange boxes indicate ORFs for auxiliary proteins, and light orange boxes highlight putative ORFs inferred from genomic sequence analysis [57].

HTLVs, positive-sense, single-stranded diploid RNA viruses, represent the first oncogenic retroviruses identified in humans [1,2,3,4]. HTLV types 1 and 2 originated independently in Africa through zoonotic transmission from non-human primates and are related to distinct lineages of simian T-lymphotropic viruses (STLVs) [20]. Both viruses can induce the transformation of primary T-lymphocytes both in vitro and in vivo. HTLV-1 is primarily associated with ATL and has also been implicated in the development of HTLV-1-associated HAM/TSP [12,30,31]. In contrast, HTLV-2 exhibits low pathogenicity and has only sporadically been linked to neurological disorders [16]. HTLV-3 and HTLV-4 have been identified in individuals involved in the hunting and consumption of bushmeat in Africa [3,20], without evidence of associated disease [22,23]. A schematic overview of the genomic organization of HTLV-1, HTLV-2, HTLV-3, and HTLV-4 is provided in Figure 1, which highlights the coding regions for regulatory (green), auxiliary (dark orange), and putative (light orange) proteins based on sequence analysis.

### 2.1. HTLV-1 and HTLV-2

HTLV-1 and HTLV-2 have a similar genomic structure, with approximately 70% of nucleotide sequence homology [19]. Both retroviruses are enveloped, with a diameter of approximately 100 nm. In vitro, HTLV-1 and HTLV-2 exhibit distinct tropism patterns. HTLV-1 primarily infects CD4+ T lymphocytes, but also CD8+ T cells, B lymphocytes, endothelial cells, and fibroblasts [32,33,34]; HTLV-2 preferentially targets CD8+ T lymphocytes [35]. Despite sharing 65% and 79% sequence identity in their envelope surface (SU) and transmembrane (TM) subunits, respectively [36], the two viruses utilize different receptor complexes for cell entry. HTLV-1 binds to heparan sulfate proteoglycans (HSPGs) and neuropilin-1 (NRP1) for initial attachment, while glucose transporter 1 (GLUT1) facilitates membrane fusion and entry. HTLV-2, although also relying on NRP1 and GLUT1, does not utilize HSPGs [37,38,39]. The expression of HTLV receptors varies among T cell subsets: HSPGs are predominantly expressed on CD4+ T cells; NRP1 is present on both CD4+ and CD8+ T cells but shows increased expression upon activation, especially in CD4+ cells; GLUT1 is expressed on both subsets and is upregulated during T-cell activation, facilitating viral entry in both CD4+ and CD8+ cells. Upon viral entry, the viral core delivers its genomic RNA along with essential enzymes, including reverse transcriptase (RT), integrase (IN), and protease (PR). While the reverse transcription process in HTLV-1 remains incompletely characterized, it is presumed to occur post-entry, leading to the synthesis of double-stranded DNA (dsDNA) [40,41]. Upon integration into the host genome, the provirus generates multiple transcripts that drive viral replication and persistence [12]. Unlike many retroviruses, HTLV-1 does not exhibit a strong preference for specific genomic integration sites [42,43,44,45]. However, in HTLV-1-associated disorders such as HAM/TSP, proviral integration occurs more frequently within transcriptionally active regions, potentially contributing to disease pathogenesis [46]. The provirus contains the promoter and enhancer elements for transcription initiation in the long terminal repeats (LTR); the polyadenylation signal for plus-strand transcription is in the $3^{\prime }$LTR. The gag, env and pol genes are expressed at the level of the LTR end sequences. Additionally, HTLV types 1 and 2 express two key proteins: Tax (Tax-1 and Tax-2) and Rex (Rex-1 and Rex-2). The Tax proteins act as potent transcriptional activators by recruiting host transcription factors [47,48], while the Rex proteins regulate the nuclear export of viral mRNAs. Rex binds to the Rex-responsive element (RexRE), enabling the selective export of regulatory transcripts during early infection and structural protein transcripts at later stages [49]. Once exported, viral mRNAs are translated by the host ribosomal machinery, with structural proteins such as Gag accumulating at the plasma membrane, where they facilitate genomic RNA packaging and virion assembly [50]. Although the intracellular trafficking of HTLV-1 Gag remains incompletely understood, evidence suggests that monomeric Gag is transported to the membrane shortly after translation [51]. The final stages of viral maturation involve proteolytic cleavage of the Gag and Pol polyproteins, yielding fully infectious virions capable of initiating new rounds of infection [52]. The proviral genomes of HTLV-1 and HTLV-2 encode the gene products of their antisense strands, HBZ and APH-2. The HTLV-1 basic leucine hinge factor (HBZ) is expressed in all ATL cell lines. HBZ is a secondary oncogene and plays a key role in cell proliferation and survival. HBZ promotes cell proliferation and survival through multiple mechanisms. It represses Tax-1 transactivation by binding to the cellular cofactors CREB and p300, thereby reducing Tax-mediated gene expression. Through its basic leucine zipper (bZIP) domain, HBZ also modulates transcription factors such as JunD, JunB, and members of the ATF/CREB family, promoting anti-apoptotic signaling and sustaining the proliferation of infected T cells. Localized in the nucleus, HBZ interferes with the interaction between Tax-1 and host transcriptional regulators, further enhancing viral persistence and contributing to oncogenesis. HBZ contains an N-terminal transactivation domain, a central modulatory domain and a C-terminal bZIP domain (responsible for its effects on JunD, JunB, c-Jun and ATF/CREB proteins). APH-2 interacts with CREB via C-terminal CREB binding and represses the transactivation of Tax-2. APH-2 does not have an activation domain and contains a canonical bZIP domain [53,54].

### 2.2. HLTV-3 and HTLV-4

In 2005, Mahieux et al. reported the identification of the human homolog HTLV-3 of STLV-3 in asymptomatic individuals from southern Cameroon. These individuals exhibited HTLV-positive serology, with serum samples originating from non-human primates (NHPs) [3,20]. In the same geographic region, HTLV-4 was also identified [3,20]. Phylogenetic analysis indicates that HTLV-4 is equidistant from HTLV-1, HTLV-2, and HTLV-3, sharing only 62–71% nucleotide identity with each [21]. The overall genomic organization of HTLV-3 and HTLV-4 is structurally analogous to that of HTLV-1 and HTLV-2, containing essential genes such as gag, pro, pol, env, tax, and rex [55]. However, the LTR sequences of HTLV-3 and HTLV-4 differ from those of HTLV-1 and HTLV-2. Notably, the LTRs of HTLV-3 and HTLV-4 possess a $5^{\prime }$ promoter region containing only two 21-bp tax-responsive elements (TREs), whereas the LTRs of HTLV-1 and HTLV-2 contain three TREs. The c-Myb binding site, responsible for recruiting the RNA polymerase III complex and interacting with Tax, is also conserved [56]. Analysis of the HTLV-4 proviral genome suggests the presence of auxiliary proteins, such as HTLV-4 ORF-IV, that share 75% sequence similarity with the HTLV-1 p13II protein, while HTLV-4 ORF-V displays low sequence similarity to the HTLV-2 p28 protein. Additionally, both HTLV-3 and HTLV-4 contain an antisense open reading frame (ORF) encoding APH-3 and APH-4 (HTLV antisense protein), analogous to HTLV-1 HBZ and HTLV-2 APH-2 [24].

## 3. Pathogenesis and Pathophysiology

Despite significant advances in elucidating HTLV-1 and HTLV-2 viral replication mechanisms and host immune responses, many aspects of their pathogenesis remain to be understood [7]. Both species (or subtypes) encode the essential structural and enzymatic proteins characteristic of retroviruses and the regulatory proteins Tax and Rex [19]. Additionally, each virus produces an RNA transcript and a corresponding protein from the negative-sense strand of the viral genome. HTLV-1 and HTLV-2 also generate multiple accessory proteins that contribute to different aspects of viral replication, evolution, and persistence [58], as reported for other viruses [59,60,61,62,63,64]. Early research on HTLV-1 infection, transmission, and pathogenesis, as well as the cellular pathways modulated by viral proteins, was largely based on studies utilizing overexpression systems in non-target cells or viral particles generated from cells transduced with the HTLV-1 proviral genome [65]. Subsequent findings demonstrated that HTLV-1 can infect various cell types in vitro and spread efficiently via cell-to-cell transmission, enabling the establishment of HTLV-1-producing cell lines—such as MT-2, MT-4, C91-PL, and SP—through co-cultivation of leukemic cells from ATL patients with human cord blood lymphocytes from healthy donors [66]. However, a major challenge in HTLV research is the lack of a reliable system to assess de novo infection, as cell-free HTLV virions exhibit minimal infectivity [67]. Over the years, numerous studies have explored the distinctions between these two viruses, with recent research particularly emphasizing differences between their regulatory proteins, Tax, and their antisense-encoded factors, HBZ (HTLV-1) and APH-2 (HTLV-2) [19]. These viral proteins engage distinct cellular pathways and signaling mechanisms, which are believed to underlie the differences in their pathogenic potential. However, several points are not fully understood, such as the precise molecular events driving tumorigenesis, the factors contributing to the progression of HTLV-1-associated leukemia to its aggressive form, and potential strategies to enhance the host immune response to prevent or delay disease onset [58].

### 3.1. Mechanisms of HTLV-1 Infection and Persistence: Virological Synapse and Clonal Expansion

HTLV-1 transmission primarily occurs through direct cell-to-cell contact rather than via the diffusion of free viral particles. Unlike many retroviruses that efficiently infect target cells in the absence of cell-cell interactions, HTLV-1 exhibits limited infectivity under such conditions. Instead, co-cultivation of virus-producing cells with permissive target cells remains the most effective means of viral spread [68]. Following the establishment of cell-to-cell contact, HTLV-1 structural proteins—such as Gag and Env—are redistributed within the infected cell, leading to the formation of a specialized interface for viral transmission known as the virological synapse (VS) [13]. Immunofluorescence and confocal microscopy studies have demonstrated that, in isolated T cells, these viral components are evenly distributed. However, upon contact with a target cell, cellular polarization directs viral proteins and genomic RNA toward the cell-cell junction [13,69]. Cryoelectron tomography studies suggest that HTLV-1 does not mediate direct plasma membrane fusion at the VS. Instead, viral particles bud from the infected cell and subsequently fuse with the target cell across this interface [13,69]. VS formation is facilitated by interactions between the adhesion molecule intercellular adhesion molecule-1 (ICAM-1) and its ligand, lymphocyte function-associated antigen-1 (LFA-1) [70,71]. Engagement of ICAM-1 activates the MEK/ERK signaling pathway, promoting the polarization of the microtubule-organizing center (MTOC) toward the VS. The viral regulatory protein Tax plays a critical role in this process by enhancing MTOC polarization and modulating ICAM-1 expression [72,73]. While Tax primarily localizes to the nucleus, it can also be detected in the cytoplasm near the MTOC and at the VS, where it contributes to signaling events that facilitate viral transmission. Notably, Tax activates the cyclic AMP response element-binding protein (CREB) pathway, leading to increased expression of Gem, a small GTP-binding protein involved in cytoskeletal remodeling and cell migration [70,74]. In addition to direct VS-mediated cell-to-cell transmission, HTLV-1 has been observed to accumulate within extracellular biofilm-like structures on the surface of infected cells [75]. These carbohydrate-rich aggregates, composed of collagen, agrin, tetherin, and galectin-3, may serve to concentrate viral particles in a confined area, thereby enhancing the likelihood of infecting susceptible target cells [75].

**Figure 2:**
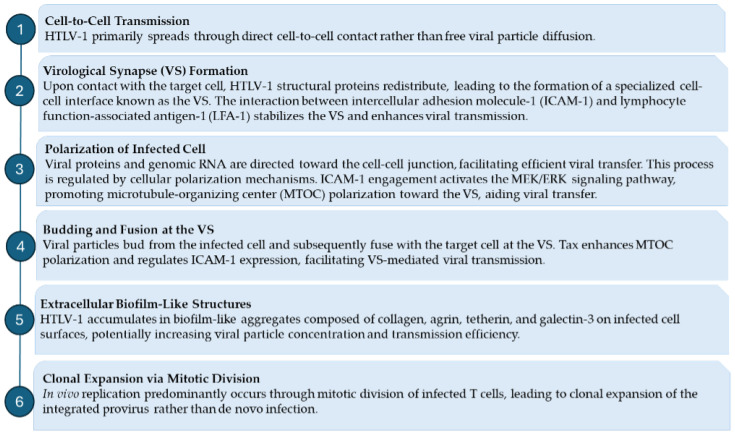
Mechanisms of HTLV-1 Infection and Persistence.

Although HTLV-1 transmission between individuals likely occurs through cell-cell contact, in vivo replication predominantly relies on the mitotic division of infected host cells, leading to the clonal expansion of the integrated provirus [76,77]. The strongest evidence supporting clonal proliferation as the primary mode of HTLV-1 propagation is the detection of clonally expanded T-cell populations in infected individuals. Despite the random integration of HTLV-1 into the host genome [78], polymerase chain reaction (PCR)-based analyses consistently reveal the presence of clonal T-cell populations in both symptomatic and asymptomatic carriers [79,80]. Given the close interplay between the molecular pathways regulating cell survival and oncogenesis, distinguishing the specific contributions of HTLV-1 to cell proliferation versus malignant transformation remains challenging. The mechanisms of HTLV-1 infection and persistence are summarized in Figure 2.

### 3.2. Differences in HTLV-1 and HTLV-2 Pathogenetic Profiles

HTLV-1 and HTLV-2 encode the transactivator proteins Tax-1 and Tax-2, which share substantial sequence similarity yet exhibit critical functional differences influencing their pathogenicity [81]. Tax-1 uniquely contains leucine zipper-like motifs that activate both canonical and non-canonical NF-κB pathways, a PDZ-binding motif (PBM) essential for PI3K/AKT/mTOR signaling, and a C-terminal secretory signal [82,83,84]. In contrast, Tax-2 has a distinctive cytoplasmic localization domain (amino acids 89–113), resulting in predominant cytoplasmic distribution, while Tax-1 localizes mainly in the nucleus [85]. Functionally, Tax-1 more strongly activates NF-κB signaling by interacting with IκB kinase components (IKKα/IKKβ/NEMO), TRAF6, and RNF8, interactions largely absent in Tax-2 [86,87,88]. Additionally, Tax-1, but not Tax-2, induces OX40 expression via non-canonical NF-κB activation [89]. Post-translational modifications also differ, with Tax-1 being more dependent on phosphorylation, ubiquitylation, SUMOylation, and acetylation for its oncogenic activity [90]. Furthermore, Tax-1, unlike Tax-2, represses p53 activity and impairs DNA damage repair, contributing to HTLV-1’s oncogenic potential [91,92].

**Figure 3:**
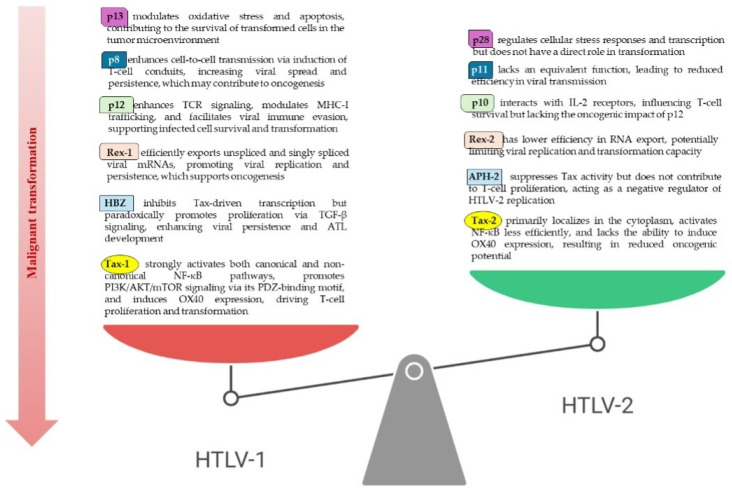
Comparison of HTLV-1 and HTLV-2 pathogenetic mechanisms.

Additionally, HTLV-1 and HTLV-2 encode different antisense-encoded factors, HBZ and APH-2 respectively. Both proteins suppress Tax-mediated proviral transcription via CREB interactions, yet HBZ exhibits stronger transcriptional inhibition, likely due to its longer half-life and unique transactivation domain [93,94]. The HBZ factor enhances TGF-β signaling, promoting viral persistence and T-cell proliferation, whereas APH-2 functions as a negative regulator of HTLV-2 replication in vivo, lacking the pro-proliferative effects of HBZ [53,95]. HTLV-1 and HTLV-2 also encode a set of regulatory and accessory proteins that contribute to their distinct pathogenic profiles. The viral RNA-binding proteins Rex-1 and Rex-2 regulate viral RNA export, yet Rex-1 shows greater efficiency in nuclear export of unspliced and singly spliced viral mRNAs [54]. The p12 protein of HTLV-1, which facilitates viral persistence by modulating T-cell receptor (TCR) signaling and MHC-I trafficking, has no direct counterpart in HTLV-2, which instead encodes p10, a protein that interacts with IL-2 receptors and influences cell survival [96,97]. Similarly, the HTLV-1 p8 protein enhances viral cell-to-cell transmission by inducing T-cell conduits, while the HTLV-2 p11 product lacks a known equivalent function [98]. The mitochondrial-targeting protein p13 of HTLV-1 modulates oxidative stress and apoptosis, while HTLV-2 expresses the functionally distinct p28, which regulates cellular stress responses and transcription [99]. Both viruses encode p30, which suppresses Tax-dependent transcription and modulates host immune evasion, yet the HTLV-1 and HTLV-2 variants differ in their post-translational modifications and interactions with cellular pathways [100]. Both HTLV-1 and HTLV-2 infections lead to the clonal expansion of T cells [79,101]. This finding implies that oligoclonal expansion does not necessarily correlate with malignant transformation. Differences in HTLV-1 and HTLV-2 pathogenetic mechanisms are summarized in Figure 3.

Understanding the mechanisms of HTLV-1 infection and persistence has significant clinical implications. The ability of the virus to spread via virological synapse and persist through clonal expansion explains the inefficacy of traditional antiviral strategies that target cell-free virions. Moreover, these processes are tightly linked to disease progression: high proviral load and dominance of certain clones have been associated with an increased risk of developing ATL and HAM/TSP. Targeting the molecular events that mediate cell-to-cell transmission, synapse formation, or clonal expansion may therefore offer promising therapeutic avenues, particularly in preventing disease onset or halting progression in early stages.

## 4. Geographical Distribution and Epidemiological Insights of HTLV

HTLV-1 exhibits a non-uniform global distribution, characterized by clusters of high endemicity situated in close proximity to regions where the virus is either absent or has a very low prevalence [102]. The primary areas where HTLV-1 is endemic or highly prevalent include Southwestern Japan, sub-Saharan Africa, South America, the Caribbean area, and specific foci in the Middle East and Australo-Melanesia [103].

In Southwestern Japan, particularly the Kyushu island and the Okinawa archipelago, HTLV-1 is highly prevalent [102]. Studies among blood donors in Japan have revealed significant variations in prevalence rates, ranging from approximately 1% in Hokkaido to over 6% in Kyushu and Okinawa, highlighting the regional clustering of the virus [102]. Sub-Saharan Africa represents a large endemic area for HTLV-1, although the precise extent of infection remains undetermined in several countries within this region [102]. The highest prevalence rates of HTLV-1 have been reported in western, central, and southern Africa, while comparatively lower rates are observed in eastern and northern Africa. Notably, in rural areas of Gabon, particularly in Haut-Ogooué, HTLV-1 seroprevalence can exceed 25% among older adults, especially women [103]. Other West African nations such as Nigeria, Ghana, and Guinea-Bissau also demonstrate significant HTLV-1 prevalence [103]. South America is another major endemic area with distinct high-prevalence zones identified in Peru (especially among Quechua populations), Colombia (Tumaco area), French Guiana (Noir-Marron population), and Brazil [104]. In Brazil, the prevalence of HTLV-1 shows heterogeneity, ranging from 0.04 to 1% among blood donors [103]. It is believed that the introduction of HTLV-1 into South America was likely associated with the historical slave trade from Africa [104]. The Caribbean area experiences high endemicity across most of its islands, with Jamaica and Haiti being particularly affected [103]. In the Middle East, foci of HTLV-1 infection have been identified in the Mashad region of Iran [103]. Australo-Melanesia also presents high prevalence, especially among Aboriginal populations in Central Australia, where rates can reach up to 44%, and in certain tribes in Papua New Guinea and the Solomon Islands, with prevalence ranging from 1.2 to 3% [103]. Additionally, southeastern regions of the USA are considered endemic for HTLV-1 [17], and a study focusing on US blood donors revealed varying prevalence rates across different regions [105]. Finally, Romania and Northeastern Iran are also recognized as endemic regions for HTLV-1 [17]. The observed geographical distribution of HTLV-1 is thought to be largely influenced by historical human migrations and the founder effect within isolated populations [102]. The higher genetic diversity of HTLV-1 in Africa supports the hypothesis that the virus originated on this continent and subsequently spread to other parts of the world through ancient human movements [17]. The concentration of the virus in specific ethnic groups and geographically isolated regions suggests that the introduction of the virus by a limited number of infected individuals, followed by sustained transmission within these communities, has played a crucial role in shaping the current global distribution.

Compared to HTLV-1, HTLV-2 exhibits a more restricted global distribution. HTLV-2 is highly prevalent among Indigenous populations of the Americas, particularly in Central and South America, including the Brazilian Amazon. In some of these communities, prevalence rates can be remarkably high, reaching up to 41.2% among the Kayapó people in Brazil [106]. Another significant population with high HTLV-2 prevalence is people who inject drugs (PWID), especially in North America and Europe [107]. In the United States, studies of blood donors have shown that HTLV-2 is more common than HTLV-1, with a higher prevalence observed among non-white individuals and those with lower levels of education [108]. HTLV-2 has also been found in some Indigenous people in the African region, including Pygmy populations in Central Africa [17]. While HTLV-1 is more prevalent among Aboriginal Australians, HTLV-2 has also been reported in this population [109]. The distinct geographical distribution of HTLV-2, with its concentration in Indigenous populations of the Americas and PWID, suggests a different historical origin and transmission dynamics compared to HTLV-1. The strong association with PWID indicates a significant role for bloodborne transmission through the sharing of needles in more recent times. The presence of HTLV-2 in some African Indigenous populations hints at either ancient connections or independent transmission events in these communities. The high prevalence among Indigenous populations in the Americas suggests an ancestral origin, possibly linked to early human migrations across the Bering Strait, followed by spread and diversification within these communities, potentially influenced by geographical isolation and specific cultural practices [109]. The later emergence and spread within PWID populations in developed countries underscore the impact of modern risk behaviors on the virus’s distribution. A summary of the geographical distribution and prevalence of HTLV-1 and HTLV-2 across different regions is provided in Table 1.

The available information on the geographical distribution of HTLV-3 and HTLV-4 is considerably less extensive compared to HTLV-1 and HTLV-2 [102]. Both viruses were discovered in 2005 in Central Africa, specifically in Cameroon [55]. HTLV-3 is recognized as the human counterpart of Simian T-cell Leukemia Virus type 3 (STLV-3), which is endemic in several monkey species inhabiting West, Central, and East Africa [55]. HTLV-4 was also discovered in the same geographical region of South Cameroon [55], and a related Simian T-lymphotropic virus type 4 has been identified in gorillas [55]. Limited evidence suggests that these viruses might also be present at low prevalence in other countries in Central and West Africa where HTLV indeterminate serologies are frequently observed [55]. However, specific studies aimed at detecting these viruses in these other regions have not yet been published. The discovery of HTLV-3 and HTLV-4 in the same geographical area where their simian counterparts are prevalent provides strong support for the hypothesis of zoonotic transmission from non-human primates to humans in Central Africa [110]. This highlights the ongoing risk of interspecies transmission of retroviruses and the potential for new human pathogens to emerge from animal reservoirs. The close genetic relationship between HTLV-3 and STLV-3, along with their overlapping geographical distribution, strongly indicates a zoonotic origin for HTLV-3 in humans. Similarly, the identification of a related virus in gorillas suggests a similar origin for HTLV-4.

### Inter-Hosts Transmission Routes

HTLV-1 and HTLV-2 are transmitted through three primary routes: exposure to infected blood or blood products, vertical transmission from mother to child, and sexual contact [27,111]. Vertical transmission via breastfeeding is the predominant mode of infection [112]. Transmission through breast milk plays a significant role in the vertical spread of HTLV, particularly HTLV-1, in many endemic regions including Japan, the Caribbean, South America and Africa [112]. The risk of mother-to-child transmission ranges from 5% to 27%, with higher rates correlated with prolonged breastfeeding duration [113,114]. While the precise mechanism by which HTLV-1 and HTLV-2 traverse the mucosal and epithelial barriers of the gastrointestinal tract remains unclear, it is hypothesized that infected lymphocytes in breast milk facilitate viral entry into the gut [115]. Once in the gastrointestinal tract, the virus may spread as free virions or through infected lymphocytes crossing the epithelial barrier. In vitro studies suggest that cell-free HTLV-1 can undergo transcytosis across the epithelium, leading to the infection of subepithelial dendritic cells, although the exact molecular mechanisms remain unresolved [116]. Additionally, it is uncertain whether HTLV-1-infected lymphocytes can transmigrate intact across the epithelial barrier to directly infect immune cells in submucosal tissue [116]. HTLV-2 can also be transmitted vertically through breastfeeding and horizontally via sexual contact; however, its most common route of spread is through the sharing of contaminated needles among intravenous drug users, particularly in North America and Europe [117,118]. The significant reduction in transmission through blood transfusions following the implementation of screening programs underscores the effectiveness of targeted public health interventions [111]. In addition to human-to-human transmission, zoonotic spillover of simian T-cell leukemia virus type 1 (STLV-1) into humans continues to be documented, particularly in Africa, where transmission occurs through contact with nonhuman primates via bites or handling of bushmeat [119,120]. Genetic analyses reveal that the viral strains detected in these individuals closely resemble STLV-1 subtypes prevalent in the primate species to which they were exposed [119,120]. Table 2 summarizes the main transmission routes of HTLV-1 and HTLV-2, along with the key populations at risk. Furthermore, the emergence of HTLV-3 and HTLV-4 has been linked to recent cross-species transmission events, with STLV-4 being endemic in African gorillas. Phylogenetic studies suggest that HTLV-4 is not an ancient human virus but rather a recent introduction into the human population through zoonotic transmission [121]. These findings underscore the role of nonhuman primates as potential reservoirs for HTLV and highlight the ongoing risk of novel HTLV variants emerging through cross-species transmission, which could have implications for viral pathogenesis and public health.

## 5. Genetics and Genomics of HTLV

### 5.1. From Viral Architecture to Host Genome Impact

The genetic and genomic landscape of Human T-lymphotropic viruses (HTLVs) provides essential insight into their ability to persist in the host, exhibit phenotypic variability, and drive oncogenesis. The HTLV genome, approximately 9 kilobases in length, includes the typical retroviral structural genes (gag, pol, env) and a regulatory pX region encoding multiple accessory proteins such as Tax and Rex, along with several minor open reading frames [122]. A notable feature is the antisense transcription that produces factors like HBZ in HTLV-1, APH-2 in HTLV-2, and other analogous proteins in emerging HTLV variants (e.g., APH-3 and APH-4), which may contribute to viral latency and immune modulation [123]. The long terminal repeats (LTRs) situated at both ends of the genome contain promoter regions with Tax-responsive elements (TREs) essential for viral gene activation [124]. Genetic diversity among HTLVs is particularly pronounced in HTLV-1, which comprises at least seven subtypes (a–g); the most widespread subtype, HTLV-1a, further divides into several regional clades [125]. HTLV-2 displays lower variability across its four subtypes. HTLV-3 and HTLV-4, considered zoonotic and rarely detected in humans, show significant divergence in their TRE regions, accessory ORFs, and LTR sequences, suggesting distinct evolutionary trajectories [126]. These differences are hypothesized to contribute to their varied, and in the case of HTLV-3 and HTLV-4, still undefined pathogenicity. A hallmark of HTLV infection is the integration of the provirus into the host genome, a process resulting from reverse transcription of viral RNA. This integration shows preference for transcriptionally active, enhancer-rich genomic regions, potentially leading to dysregulation of nearby genes or activation of oncogenes [127]. The infection is characterized by clonal expansion of T cells harboring identical integration sites, a phenomenon observed even in asymptomatic carriers and particularly associated with HTLV-1 and HTLV-2 [128]. The regulation of proviral gene expression is further modulated by epigenetic mechanisms. DNA methylation and histone modifications—especially the methylation or deacetylation of histone marks H3K9 and H3K27—are associated with silencing of the sense promoter, contributing to viral latency [129]. In contrast, the antisense promoter, particularly for HBZ, often remains active, supporting long-term persistence of infected clones. This transcriptional asymmetry may underlie mechanisms of immune escape and malignant transformation. Therapeutic approaches aimed at reactivating latent provirus through epigenetic modulation (e.g., “shock-and-kill” strategies) are currently under investigation [130]. HTLV proviral transcription exhibits a dynamic pattern, alternating between phases of active expression—particularly for sense transcripts like Tax and Rex—and latency. The antisense transcript, such as HBZ, maintains more consistent expression, suggesting a central role in maintaining viral persistence and modulating the host immune environment [131]. Experimental models have demonstrated that altering HBZ levels can affect the proliferation of infected T cells and their responses to oxidative stress [132]. Recent technological advances are accelerating the understanding of HTLV biology. Genome-editing tools like CRISPR/Cas9 have been used to target and disrupt integrated proviral sequences, notably Tax and HBZ, thereby reducing viral expression and clonal proliferation in cellular models, although in vivo delivery and specificity remain limitations [133]. Epigenomic techniques such as ATAC-seq and ChIP-seq, along with single-cell RNA sequencing, have enabled the identification of distinct epigenetic landscapes and clonal features associated with transformation risk [134]. Furthermore, integrating single-cell data with T-cell receptor (TCR) clonotype and methylation profiles has revealed subpopulations predisposed to malignancy [135]. In such a context, Artificial intelligence and machine learning approaches are increasingly being employed to predict proviral integration sites, stratify infection risk, and classify novel viral isolates. Deep learning algorithms have improved functional annotation of HTLV sequences, enhancing the precision of bioinformatic analyses in both clinical and research contexts [136]. HTLV-3 and HTLV-4, although structurally similar to HTLV-1 and HTLV-2, exhibit distinct genomic characteristics. In HTLV-3, the tax gene shows functional homology with that of HTLV-1, though its expression appears reduced due to differences in LTR promoter efficiency. HTLV-4, more distantly related, encodes unique accessory ORFs (e.g., ORF-IV, ORF-V) whose functions remain speculative. Both viruses also express antisense transcripts, such as APH-3 and APH-4, suggesting that bidirectional regulation is a conserved feature potentially relevant for latency control even in viruses without known pathogenicity [24].

### 5.2. Beyond Viral Architecture: HTLV Integration as a Source of Genomic Instability

Beyond the viral genome structure and regulatory mechanisms, recent research has begun to investigate the broader implications of HTLV integration on host genomic integrity. The following section explores how HTLV proviruses may contribute to genome instability and chromatin disruption in infected cells. The integration of retroviral DNA into host genomes is a defining feature of deltaretroviruses such as HTLV. While the biological consequences of proviral expression and clonal expansion have been extensively characterized, the potential for HTLV integration to induce genomic instability in host cells remains an underexplored but increasingly relevant field. Unlike insertional mutagenesis observed in gammaretroviruses, HTLV-1 and HTLV-2 typically integrate into transcriptionally active regions without strong site specificity. However, recent high-throughput mapping technologies have revealed recurrent integration events near proto-oncogenes and regulatory elements, suggesting that HTLV may exert subtle but chronic influences on chromatin architecture and gene expression fidelity [137]. In particular, proviral integration near super-enhancers or within topologically associating domains (TADs) may disrupt three-dimensional genome organization, altering the transcriptional output of distal genes through enhancer hijacking or boundary insulation loss [137]. Such mechanisms are increasingly recognized as oncogenic drivers in other contexts, such as in leukemia associated with MLL rearrangements [138]. In the case of HTLV-1, integrated proviruses have been shown to colocalize with actively transcribed regions rich in histone marks H3K27ac and H3K4me1, supporting the hypothesis that HTLV-1 may reshape host chromatin topology [139]. Another area of concern involves defective proviruses—integrated viral genomes that have lost the capacity to express complete viral transcripts due to deletions or hypermutations [140]. Though replication-incompetent, these sequences can still harbor strong promoters and enhancers capable of cis-acting effects on adjacent genes, as observed in endogenous retroviruses (ERVs) [141]. Preliminary data suggest that HTLV-derived long terminal repeats (LTRs) retain transcriptional activity and may act as cryptic promoters in transformed T cells, potentially contributing to aberrant transcriptional landscapes in ATL [142]. Moreover, the process of integration itself may induce DNA damage and chromosomal rearrangements. Although HTLV integrase does not generate double-stranded breaks in the classical sense, the recruitment of host DNA repair machinery during the integration process may result in error-prone repair or replication stress, particularly when occurring in fragile genomic regions [143]. This possibility is reinforced by the presence of DNA damage markers such as γH2AX and ATM activation in HTLV-infected cell lines [144]. From a therapeutic perspective, understanding the mutagenic potential of HTLV integration opens new avenues for targeted interventions. For example, mapping integration sites at the single-cell level using long-read sequencing technologies could identify high-risk clones prior to malignant transformation [145]. Furthermore, epigenetic therapies may offer a means of mitigating the transcriptional impact of proviral integration without directly targeting viral genes, especially in cases where the provirus is silent but still exerts oncogenic influence through its regulatory elements. In other words, while HTLV-induced transformation has traditionally been attributed to viral protein function and clonal expansion, mounting evidence points to a broader genomic influence exerted through integration events. Future research should focus on unraveling the interplay between proviral DNA, chromatin remodeling, and genome topology in order to fully elucidate the role of HTLV in genomic instability and leukemogenesis.

## 6. Diagnosis of HTLV Infections

Accurate and timely diagnosis of HTLV infections is fundamental for effective clinical management, public health surveillance, and expanding our understanding of HTLV-associated diseases. While HTLV-1 and HTLV-2 are the most extensively studied due to their associations with ATL and HAM/TSP, the clinical relevance of the more recently discovered HTLV-3 and HTLV-4 remains uncertain. Given the similarities in transmission routes and genetic structures, distinguishing among these viruses requires a multi-layered diagnostic approach [146].

The diagnostic workflow typically begins with serological screening. The Enzyme-Linked Immunosorbent Assay (ELISA) is the most widely used initial test, detecting antibodies against viral proteins—particularly the envelope (gp46) and core (p24) antigens. Although ELISA is highly sensitive, its specificity can be reduced due to cross-reactivity with other retroviruses such as HIV, leading to false-positive results, especially in regions with low HTLV prevalence [147,148]. Thus, ELISA-reactive samples should always be subjected to confirmatory testing.

Confirmatory assays include Western Blot (WB) and Line Immunoassay (LIA), which assess reactivity to specific HTLV proteins such as gp21, gp46, p19, and p24 [149,150]. These methods also allow differentiation between HTLV-1 and HTLV-2 [151,152]. WB, for example, demonstrates a sensitivity of 97.1% and specificity of 97.5% [153]. However, indeterminate patterns—due to partial reactivity or low antibody titers—can complicate interpretation and necessitate molecular testing.

A more recent addition to the diagnostic techniques is the Chemiluminescent Microparticle Immunoassay (CMIA), which provides high-throughput automation and enhanced specificity [154]. CMIA is especially useful for large-scale screening, such as in blood banks, due to its reduced turnaround time and minimal manual handling [150]. However, while CMIA is effective for detecting HTLV infections, it does not differentiate between HTLV-1 and HTLV-2, necessitating follow-up with WB, LIA, or molecular assays.

Molecular diagnosis plays a central role in confirming HTLV infections. Polymerase Chain Reaction (PCR) is the gold standard for detecting proviral DNA, offering qualitative and quantitative capabilities [154,155,156]. Quantitative PCR (qPCR) is particularly valuable for monitoring proviral load, which correlates with disease progression, particularly in ATL and HAM/TSP [157]. RT-PCR may be used to detect viral RNA in plasma, although its clinical use is limited due to low RNA levels in circulation [158]. Nested PCR, using sequential amplifications, improves detection in low-proviral-load contexts.

Subtype differentiation is essential for guiding clinical management and public health interventions. Subtype-specific PCR uses primers targeting conserved genetic regions such as tax and LTR for HTLV-1, and tax and pol for HTLV-2. HTLV-3 and HTLV-4 are identified through unique tax sequences [146]. These assays are highly specific and allow for precise classification.

For even greater resolution, Next-Generation Sequencing (NGS) offers comprehensive analysis of HTLV genomes, enabling the identification of mixed infections, intra-strain diversity, and viral evolution [159,160]. Despite its diagnostic power, NGS remains primarily in the research domain due to cost and technical requirements.

Beyond conventional diagnostics, biosensor-based methods are emerging as promising alternatives. These devices employ biological receptors and electronic transducers to detect HTLV DNA or antigens with high sensitivity [161]. Materials like graphene and polypyrrole enable miniaturization and low-cost production [162,163,164], making biosensors suitable for point-of-care applications [165,166].

Additional tools include flow cytometry, which detects immunophenotypic shifts in HTLV-infected cells [167,168], and cerebrospinal fluid (CSF) analysis in cases of suspected HAM/TSP. Diagnostic markers in CSF include pleocytosis, elevated HTLV-1 intrathecal antibody index, and oligoclonal bands [169].

### Challenges and Future Directions in HTLV Diagnosis

Despite the advancements in HTLV diagnostics, several challenges remain. One of the most significant issues is serological ambiguity, which necessitates confirmatory molecular testing. Additionally, HTLV-3 and HTLV-4 are rarely detected, and the lack of commercially available diagnostic assays for these subtypes hampers research and epidemiological tracking. Furthermore, the clinical implications of proviral load variability in HTLV-3 and HTLV-4 infections are not yet fully understood (Table 3).

Looking to the future, research is focused on improving diagnostic accessibility and accuracy. The development of point-of-care testing for HTLV would allow for rapid diagnosis in resource-limited settings, particularly in endemic regions. Moreover, advancements in NGS-based surveillance could facilitate the detection of novel HTLV variants. Another promising area is the application of artificial intelligence (AI) in HTLV diagnostics, where machine learning algorithms could enhance the interpretation of serological and molecular data, reducing diagnostic uncertainties [136,170].

## 7. Clinical Management of HTLV Infections

HTLVs have been associated with various clinical conditions, ranging from asymptomatic infections to severe hematological and neurological diseases. Among these, HTLV-1 and HTLV-2 have been extensively studied, while HTLV-3 and HTLV-4 remain largely enigmatic, with no definitive disease associations identified so far. HTLV-1 is the only type with clearly established pathogenicity, causing severe malignancies (ATL) and neuroinflammatory disorders (HAM/TSP) [171,172]. HTLV-2 remains controversial, with potential but unconfirmed associations with neurological and immune disorders [173]. HTLV-3 and HTLV-4 are recently discovered and require further epidemiological studies to determine any pathogenic role in humans (Table 4).

### General Clinical Management Principles

The clinical management of HTLV infections is multifaceted, requiring a thorough understanding of the virology, pathogenesis, and therapeutic options available for infected individuals. Most individuals infected with HTLV-1 or HTLV-2 remain asymptomatic throughout their lifetime, and in such cases, clinical management focuses primarily on monitoring, risk reduction, and patient education [174]. Periodic follow-up, including routine clinical evaluation and laboratory assessments, is recommended to detect early signs of disease progression, particularly in those with high proviral loads, which have been correlated with an increased risk of developing HTLV-associated conditions. Counseling plays an essential role in preventing transmission, particularly in endemic regions. Patients are advised to engage in safe sexual practices, avoid breastfeeding if they are HTLV-1-positive, and refrain from donating blood, organs, or tissues.

Although antiviral therapies for HTLV infections remain largely investigational, several agents have been explored for their potential to suppress viral replication. Nucleoside reverse transcriptase inhibitors such as zidovudine (AZT) [175] and lamivudine (3TC) have been studied [176,177], although their clinical efficacy in halting disease progression remains uncertain. Similarly, the use of tenofovir (TDF) [178] was analyzed. Integrase inhibitors such as raltegravir (RAL) [179,180,181] have demonstrated some potential in reducing proviral loads, but their impact on disease outcomes is still under investigation. Interferon-alpha (IFN-α), known for its immunomodulatory properties, has shown some efficacy in combination with AZT, particularly in patients with HTLV-associated malignancies [175,182].

Antiretroviral therapy (ART) in HTLV-infected individuals has produced heterogeneous and often inconclusive outcomes. While some studies have reported transient reductions in proviral load (PVL), these effects are generally not sustained over time, indicating limited long-term virological benefit [182]. Immunological responses to ART also appear inconsistent: in certain cohorts, ART is associated with a decrease in lymphocyte counts, whereas in others, CD4^+^ and CD8^+^ cell populations remain stable or even increase, suggesting patient-specific variability or methodological differences among studies.

Clinically, reported benefits are largely anecdotal or subjective. Some patients describe improvements in symptoms such as neuropathic pain or gait disturbances; however, these effects are not consistently corroborated by objective neurological or functional assessments. To date, no ART regimen has demonstrated clear efficacy in reversing or halting neurological deterioration in HTLV-associated diseases such as HAM/TSP or ATL. Given the variability of clinical and virological responses, current evidence does not support the routine use of ART as a standard treatment for HTLV-1 or HTLV-2 infections outside of specific clinical trials or ATL-associated contexts. Further research—particularly studies employing standardized treatment protocols, rigorous clinical endpoints, and larger patient cohorts—is essential to better define the potential role of ART in HTLV management.

Howoever, the access to diagnosis and treatment of HTLV infections remains a significant challenge in many endemic regions, particularly in low- and middle-income countries (LMICs) such as parts of Latin America, sub-Saharan Africa, and the Caribbean. In these settings, diagnostic capabilities are often limited due to the high cost and technical requirements of confirmatory tests, such as Western Blot, Line Immunoassay, and PCR-based methods. Recently, a nested real-time PCR technique followed by high-resolution melting (rtPCR-HRM) has been proposed as an inexpensive and effective molecular diagnostic tool for simultaneous identification of HTLV-1 and HTLV-2, which is particularly useful in Latin American countries where both viruses circulate [183].

Many health systems in endemic areas lack the necessary infrastructure for widespread screening, leading to underdiagnosis and delayed clinical management. For example, in Nigeria, the prevalence of HTLV-1/2 among pregnant women has been underestimated due to the lack of systematic screening programs [184]. In addition, treatment options, especially those involving multidisciplinary care for HTLV-1-associated diseases such as ATL and HAM/TSP, are frequently centralized in urban tertiary centers, creating barriers for rural populations. Management of ATL, for example, requires complex therapeutic approaches that are not widely available globally.

Economic restrictions and limited public awareness further exacerbate the problem. In some countries, HTLV is not included in national screening programs for blood donation or prenatal care, despite its potential for vertical and horizontal transmission. Although programs in Japan and the United States have been successful in interrupting the spread of HTLV-I through transfusions, such initiatives are not universally implemented [185]. In addition, antiretroviral therapies, immunomodulatory drugs, and symptomatic treatments are often not covered by national health insurance systems, making long-term management of the disease unaffordable for many patients. Currently, treatment options for HTLV-1 are limited and focus primarily on symptom management, with few curative interventions available [171].

Addressing these disparities requires international support, increased investment in public health infrastructure, and the development of affordable diagnostics and therapies. For example, the use of dried blood samples (DBS) has proven useful in the diagnosis and virological characterization of retrovirus infections in resource-limited settings [186].

## 8. Disease-Specific Management Strategies

HTLV-1 is primarily associated with ATL and HTLV-1-associated myelopathy/tropical spastic paraparesis (HAM/TSP), both of which require specialized treatment approaches tailored to disease severity and patient-specific factors. Additionally, HTLV-1 has been implicated in inflammatory disorders, including HTLV-1-associated uveitis, polymyositis, and alveolitis, which necessitate distinct management strategies.

### 8.1. Management of ATL

ATL is an aggressive CD4+ T-cell malignancy that manifests in four clinical subtypes: acute, lymphoma, chronic, and smoldering. The therapeutic approach varies depending on the subtype, with aggressive ATL (acute and lymphoma forms) requiring more intensive treatment than indolent ATL (chronic and smoldering forms [146]). Currently, there is an urgent need to develop novel therapies for ATL to improve survival outcomes. Advances in the molecular and epigenetic landscape of ATL, along with early disease detection, offer opportunities for early intervention and personalized treatment strategies. Understanding the molecular genetics of ATL has provided critical insights into its pathogenesis and potential therapeutic targets. A key step in improving patient outcomes is the identification of asymptomatic carriers at high risk of progression to ATL. Traditionally, this has been based on quantifying the PVL. However, recent studies suggest that assessing oligoclonality—particularly the expansion of specific clones—using molecular and flow cytometric techniques provides a more accurate risk assessment. These approaches have demonstrated that carriers with oligoclonal populations are at an increased risk of disease transformation, beyond what PVL alone can predict, highlighting the potential for improved risk stratification and earlier therapeutic intervention [146].

For aggressive ATL, standard therapy involves combination chemotherapy, typically CHOP-like regimens (cyclophosphamide, doxorubicin, vincristine, prednisone), which are frequently used as first-line treatment [187]. However, ATL cells exhibit intrinsic resistance to conventional chemotherapy, contributing to high relapse rates and poor overall survival [188,189]. In leukemic subtypes, the combination of zidovudine (AZT) and interferon-alpha (IFN-α) has emerged as a promising alternative, demonstrating improved long-term survival in select patient populations, particularly those with non-lymphomatous ATL [190]. Overall, the addition of antivirals to chemotherapy can extend one-year progression-free survival and one-year overall survival in patients with Adult T-cell Leukemia/Lymphoma. Specifically, patients treated with chemotherapy in combination with antivirals exhibited lower tumor and viral loads, reduced white blood cell counts, decreased lactate dehydrogenase and β2-microglobulin levels, and a lower Ki-67 positive rate compared to those receiving chemotherapy alone [190]. However, the efficacy of this regimen is influenced by factors such as disease burden, patient age, and immune status.

Intensive chemotherapy alone is insufficient to prevent relapse in aggressive ATL. Allogeneic hematopoietic stem cell transplantation offers a promising curative option for younger patients, with reduced-intensity conditioning regimens lowering mortality rates and improving donor availability. Furthermore, new therapies like mogamulizumab, brentuximab vedotin, tucidinostat, and valemetostat have recently emerged for treating aggressive ATL [191].

For relapsed or refractory ATL, targeted therapies have gained increasing attention. Mogamulizumab, a monoclonal antibody against CC chemokine receptor 4 (CCR4), has shown significant efficacy in depleting ATL cells, particularly in patients expressing CCR4 on malignant T cells [192,193,194,195,196,197].

A phase 2 study evaluating this monoclonal antibody in patients with relapse or refractory (R/R) aggressive ATL demonstrated an overall response rate (ORR) of 50% and median progression-free survival (PFS) of 5.2 months [192]. In a subsequent randomized phase 2 trial for newly diagnosed patients, the combination of mogamulizumab with VCAP-AMP-VECP increased the complete response (CR) rate to 52% compared to 33% with VCAP-AMP-VECP alone, although no significant survival differences were observed between the regimens [193,195].

A retrospective analysis of transplant-ineligible patients indicated that those receiving mogamulizumab had a four-year overall survival (OS) probability of 46.3% and a median survival time (MST) of 36.1 months, in contrast to 20.6% OS and 7.8 months MST for the chemotherapy-only group. Most patients were treated with the less toxic CHOP regimen due to the higher toxicity of VCAP-AMP-VECP, particularly in older individuals [197].

Genomic studies suggest that gain-of-function mutations in CCR4 may predict sensitivity to mogamulizumab, while alterations in TP53 and CD274 correlate with poorer survival outcomes. Further research is required to identify patients who will benefit most from this treatment [194,196].

The use of mogamulizumab prior to allogeneic hematopoietic stem cell transplantation (allo-HSCT) has been associated with an increased risk of severe steroid-refractory acute graft-versus-host disease (GVHD), with incidence rates of 30.9% for grade 3–4 acute GVHD and 48.9% for steroid-refractory GVHD among treated patients compared to 17.2% and 23.5% in the untreated cohort, respectively. Administering mogamulizumab within 50 days of allo-HSCT raises nonrelapse mortality (NRM) risks. Post marketing surveillance has shown that the occurrence of acute GVHD is significantly higher in patients receiving allo-HSCT within 90 days of their last mogamulizumab dose [198]. In patients relapsed after allo-HSCT, mogamulizumab may induce immune-mediated anti-tumor effects. The Nagasaki transplant group reported that three out of nine relapsed patients achieved CR without disease progression or GVHD, while a retrospective study indicated rapid disappearance of ATL cells in peripheral blood following treatment, although lymph node lesions showed resistance [199,200].

Other targeted approaches, including lenalidomide (an immunomodulatory agent) [201,202] and bortezomib (a proteasome inhibitor) [191,203,204], are being explored for their potential to disrupt ATL survival pathways. Several treatments have been developed for relapsed/refractory aggressive ATL, including mogamulizumab, brentuximab vedotin, tucidinostat, lenalidomide and valemetostat. However, there is currently no evidence demonstrating the superiority of any of these agents in terms of overall response rate or overall survival.

For eligible patients, allo-HSCT remains the only potentially curative therapy for ATL. Improved donor availability has enhanced access to transplants, and the use of reduced-intensity conditioning (RIC) regimens has lowered transplant-related mortality, resulting in extended survival for these patients. HSCT is particularly considered in younger patients with chemosensitive disease, though its application is limited by high treatment-related morbidity and mortality, as well as the risk of graft-versus-host disease (GVHD).

Patients with aggressive ATL often have poor prognosis without treatment that includes allo-HSCT. For those who respond to first-line chemotherapy, allo-HSCT is advised for patients under 70 years with sufficient organ function. According to the modified ATL-PI, the VCAP-AMP-VECP regimen is recommended over the CHOP regimen as the preferred induction therapy for transplant-eligible patients, especially in intermediate- and high-risk populations [205]. Early transplantation within 100 days of diagnosis may enhance survival [206]. Allo-HSCT can potentially cure some patients with aggressive ATL, but high treatment-related mortality is a significant concern. Retrospective studies indicate that overall survival and relapse rates with reduced-intensity conditioning (RIC) are comparable to those with myeloablative conditioning, making RIC particularly advisable for patients aged 50 and older [207,208,209].

For patients without suitable HLA-matched donors, cord blood transplantation and haploidentical HSCT (haplo-HSCT) provide viable alternatives. Although CBT initially showed high treatment-related mortality rates (10–46%), later studies demonstrated better non-relapse mortality and survival outcomes comparable to related or unrelated donors [210,211,212].

Haplo-HSCT with post-transplant cyclophosphamide has also yielded favorable results. A phase 1/2 trial for aggressive ATL noted overall survival rates of 83% at one year and 73% at two years, with non-relapse mortality and disease progression rates of 11% and 28% at one year, respectively [213].

### 8.2. Management of HAM/TSP

HAM/TSP is a chronic progressive myelopathy characterized by spastic paraparesis, bladder dysfunction, and sensory disturbances, resulting from HTLV-1-induced inflammation in the central nervous system. HAM/TSP management requires a comprehensive multidisciplinary approach integrating disease-modifying therapies, symptomatic treatments, and rehabilitation strategies. Disease-modifying approaches primarily focus on modulating the aberrant immune response characteristic of HAM/TSP pathophysiology. Corticosteroids remain a cornerstone of immunomodulatory treatment, with pulse methylprednisolone therapy (1g/day for 3–5 days) demonstrating significant temporary improvement in motor function scores and reduction in inflammatory markers in cerebrospinal fluid [214]. Most patients experience meaningful but transient clinical benefits, with effects typically waning within 1–3 months, necessitating consideration of maintenance therapy [215]. Oral prednisolone (0.5–1 mg/kg/day) represents a common maintenance strategy, though long-term administration is limited by adverse effects including osteoporosis, hyperglycemia, and increased infection susceptibility [215,216]. The immunomodulatory benefits of corticosteroids must be balanced against these potential complications, particularly in patients requiring prolonged therapy.

Interferon-α therapy has demonstrated promising results in modulating the aberrant immune response in HAM/TSP. A pivotal randomized controlled trial established that interferon-α (3 million units three times weekly for 4 weeks) led to significantly improved motor function scores and decreased HTLV-1 proviral load compared to placebo [217]. Subsequent analysis has identified disease duration as a critical factor in treatment response, with patients in earlier disease stages (less than 4 years from symptom onset) showing more robust clinical improvement [218]. This finding underscores the importance of early intervention in HAM/TSP management. Common adverse effects of interferon therapy include flu-like symptoms, depression, and transient cytopenias, necessitating careful monitoring throughout the treatment course [217,218].

Various other immunomodulatory approaches have been investigated with variable efficacy. A small but notable trial evaluating cyclosporine A in steroid-refractory HAM/TSP patients demonstrated modest improvements in spasticity and gait parameters in approximately 60% of participants [219]. Tacrolimus, another calcineurin inhibitor, showed potential benefit in a retrospective case series, particularly for pain and spasticity reduction [220]. More recently, biological agents targeting specific immune pathways have shown promise. Mogamulizumab, a CCR4-targeting monoclonal antibody that depletes HTLV-1-infected cells, has demonstrated encouraging results in reducing spasticity and improving urinary symptoms in a phase I/II trial [221]. However, concerns regarding potential acceleration of other autoimmune conditions have tempered enthusiasm and necessitate careful patient selection [221,222].

Effective management of spasticity significantly improves mobility and quality of life for HAM/TSP patients. Baclofen, a GABA-B receptor agonist (typically initiated at 5 mg three times daily and titrated based on clinical response), represents first-line therapy for HAM/TSP-related spasticity. A comparative study investigated the efficacy of baclofen in spasticity, comparing it with tolperisone in patients with spinal cord injury. The results showed a significant reduction in spasticity in patients treated with baclofen, with improvements in Ashworth scale scores and muscle strength, suggesting a potential benefit for patients with HAM/TSP [223]. When baclofen is poorly tolerated or ineffective, alternative agents such as tizanidine or benzodiazepines may be considered. Recent studies have shown that tizanidine can offer similar antispastic effects while being better tolerated in some cases [224]. For patients with severe, refractory spasticity, intrathecal baclofen therapy has demonstrated substantial improvement in mobility and a significant reduction in pain scores, as evidenced by a recent prospective observational study [225]. Additionally, targeted botulinum toxin injections are a promising approach for managing focal spasticity, especially when it affects gait biomechanics or contributes to painful spasms, with recent research showing notable reductions in spasticity and pain in patients post-stroke [226].

Neurogenic bladder dysfunction affects up to 90% of patients with HAM/TSP and significantly affects quality of life. Urodynamic studies are critical to characterize patterns of dysfunction and guide treatment. A retrospective analysis of 60 patients with HAM/TSP found that irritative symptoms, such as urgency and frequency, were the most common, with a prevalence of 75%, and that detrusor-sphincter dyssynergy was present in 78% of cases [227]. Antimuscarinic agents, such as oxybutynin and solifenacin, are used to manage detrusor overactivity. An updated systematic review confirmed the efficacy of solifenacin in reducing episodes of urgency and incontinence, urinary frequency, and nocturia, with increased urinary volume over 24 h compared with placebo [228]. However, an increase in side effects was observed compared with placebo, underscoring the need for clinical monitoring. Clean intermittent catheterization is indicated in patients with significant residual postminctional volumes (>100 mL) or detrusor-sphincter dyssynergy. A prospective study of 547 patients showed that initiation of catheterization led to significant improvement in urinary symptom scores (HAM-BDSS) [229]. Alpha-adrenergic antagonists may be useful in patients with obstructive components of voiding. One review suggested that alpha-blockers may have a modest but useful effect in facilitating retention and emptying, and in preventing autonomic dysreflexia [230].

Comprehensive rehabilitation programs play a crucial role in the management of HTLV-1 syndrome (HAM/TSP). A randomized controlled clinical trial showed that a 12-week structured rehabilitation program, including strength training exercises, gait training, and balance improvement, led to significant improvements in patients’ functional independence scores and quality of life compared with standard care. This type of intervention, which combines several therapeutic modalities, not only improves patients’ mobility, but also has positive effects on self-sufficiency and daily activity management, reducing the risk of long-term disability. Program participants showed a significant increase in functional independence and a reduction in fatigue, a debilitating symptom for patients with HAM/TSP. The observed improvements were evident in all areas of motor function, with particular emphasis on the ability to walk, perform daily movements and maintain good balance during daily activities [231]. In addition, regular physiotherapy is essential to optimize mobility and prevent complications associated with prolonged immobility, such as muscle contractures, atrophy, and increased fall risk. Occupational therapy plays a crucial role by focusing on a thorough assessment of daily activities, with special attention to energy conservation techniques and the use of assistive technologies to facilitate activities such as eating, dressing, and personal care. Studies have shown that assistive technologies (AT), such as motorized wheelchairs and walking support devices, significantly improve patients’ autonomy and help prevent further deterioration of their physical condition. For example, a study examined the effectiveness of occupational therapy based on AT to improve occupational performance, satisfaction, and psychosocial impacts in individuals recovering from a stroke. The results showed significant improvements in occupational performance, satisfaction, and goal attainment in meaningful activities, as measured between pre-test, post-test, and follow-up assessments. User satisfaction with AT and associated services was generally reported as ’satisfactory’, with a significant increase in average scores from post-intervention to follow-up. Additionally, the psychosocial impact of assistive technologies significantly improved from post-intervention to follow-up evaluation. These findings underscore the positive role of assistive technologies in enhancing functional outcomes and overall well-being in rehabilitation [232]. A systematic review also examined the effectiveness and feasibility of early physical rehabilitation programs for hospitalized geriatric patients. The findings suggested that early rehabilitation programs, particularly those that are multidisciplinary and include exercise components, can improve independence in activities of daily living (ADLs) and facilitate a return to home. However, specific information regarding the intensity, duration, and frequency of exercises is often lacking. This highlights the need for more standardized approaches in designing early rehabilitation programs for the elderly, ensuring that they are both effective and sustainable in promoting long-term functional recovery [233].

The profound impact of HAM/TSP on quality of life goes beyond physical disability. One study identified that pain control, preservation of mobility, and bladder management are the three domains most strongly correlated with overall quality of life in patients with HAM/TSP [234]. This finding underscores the importance of comprehensive symptom management in addition to disease-modifying approaches. Psychological support should be routinely incorporated into management plans for patients with HAM/TSP, as depression has been consistently documented to have a significant impact on both quality of life and treatment adherence. A cross-sectional study of 108 people with HTLV-1 infection, including 47 with HAM/TSP, found a prevalence of depression of 37.96%, with a significant association between the disease and depression, particularly in patients aged 18–39 years [235]. In addition, a systematic review reported an overall prevalence of depression of 35% (95% CI: 27–43%) among individuals with HTLV-1 infection, highlighting that depression is particularly prevalent among patients with HAM/TSP [236]. These findings underscore the critical need to address psychological factors as part of comprehensive disease management.

The management of HAM/TSP remains a complex challenge that requires a multidisciplinary approach and the integration of disease-modifying therapies, symptomatic treatments, and rehabilitation strategies. Although current therapeutic approaches, such as corticosteroids and interferon-α, have shown positive results in some cases, long-term effects are limited and the need for maintenance therapies is evident. Combinations of antiretroviral therapy, such as raltegravir and zidovudine, have shown modest benefit, but with inconsistent clinical responses. On the other hand, emerging approaches in regenerative medicine, such as mesenchymal stem cell transplantation, offer a promising opportunity to modulate inflammation and improve motor function, although the safety and efficacy of these therapies have yet to be confirmed through larger clinical trials. Integration of innovative therapies and continued evaluation of new treatments are critical to improving the quality of life of patients with HAM/TSP. Further clinical and preclinical studies are needed to identify and validate more effective and sustainable long-term therapeutic approaches.

### 8.3. Management of HTLV-1-Associated Inflammatory Disorders

HTLV-1 is also implicated in various immune-mediated inflammatory diseases, including HTLV-1-associated uveitis, polymyositis, and alveolitis.

- HTLV-1-associated uveitis: Chronic intraocular inflammation that may lead to vision impairment or blindness. Treatment includes topical, periocular, or systemic corticosteroids, with immunosuppressive agents (e.g., cyclosporine, methotrexate) reserved for refractory cases. Regular ophthalmologic evaluations are necessary to monitor disease activity and prevent complications [237,238,239,240].
- HTLV-1-associated polymyositis: An inflammatory myopathy characterized by progressive muscle weakness and elevated creatine kinase levels. Corticosteroids and immunosuppressants are commonly used, with physical therapy playing a key role in muscle function preservation [241,242,243].
- HTLV-1-associated alveolitis: A pulmonary inflammatory condition associated with interstitial lung disease and respiratory dysfunction. Treatment typically includes corticosteroids and bronchodilators, though disease progression can lead to fibrotic lung damage in severe cases [244,245].

Overall, the management of HTLV-1-associated diseases requires a multidisciplinary approach, incorporating antiviral, immunomodulatory, and supportive therapies to optimize patient outcomes. As research progresses, novel targeted therapies and immunotherapies hold potential for improving survival and quality of life in affected individuals.

### 8.4. Emerging Therapies and Future Directions

Recent advances in biomedical research have led to the development of novel therapeutic strategies for HTLV-associated diseases, particularly adult T-cell leukemia/lymphoma and HTLV-1-associated myelopathy/tropical spastic paraparesis, as summarized in Table 5. Given the persistence of HTLV infection and the lack of definitive curative therapies, current research is focused on innovative approaches, including gene-editing technologies, immunotherapeutic strategies, and targeted pharmacological interventions.

One of the most promising avenues involves gene-editing technologies, particularly CRISPR-Cas9 and zinc finger nucleases (ZFNs), which are being investigated for their potential to selectively excise HTLV-1 proviral DNA from infected host genomes [246,247]. By disrupting viral genes essential for replication and transformation, these approaches aim to eliminate latent reservoirs and prevent oncogenesis. However, significant challenges remain regarding delivery efficiency, off-target effects, and long-term safety. Research is currently focused on improving vector-based delivery systems such as lipid nanoparticles and viral vectors, as well as exploring ex vivo gene-editing approaches in hematopoietic stem cells (HSCs) as a potential means of achieving viral eradication.

Another rapidly evolving area is immunotherapy, particularly immune checkpoint inhibitors and chimeric antigen receptor (CAR) T-cell therapy [248]. Given the role of immune evasion in ATL pathogenesis, inhibitors targeting the PD-1/PD-L1 axis, such as nivolumab and pembrolizumab, are being evaluated for their ability to enhance anti-tumor immune responses. However, their clinical application is complicated by the risk of immune-related toxicities. CAR-T therapy, which involves engineering T cells to express antigen-specific chimeric receptors, is emerging as a potential treatment for relapsed/refractory ATL. Constructs targeting CD4 and CD25 have demonstrated preclinical efficacy, though further optimization is required to reduce off-target cytotoxicity and improve persistence. In addition to immunotherapy, novel targeted therapies have been developed for ATL. The anti-CCR4 monoclonal antibody mogamulizumab has been approved in Japan and has demonstrated efficacy in depleting ATL cells and reducing viral burden. Other agents, such as lenalidomide, have shown promise in modulating the immune response and inducing apoptosis in HTLV-infected cells, particularly in smoldering and chronic ATL subtypes. Moreover, proteasome inhibitors like bortezomib are being explored for their ability to disrupt NF-κB signaling, which is critical for ATL cell survival. For HAM/TSP, treatment remains largely symptomatic and aimed at slowing disease progression and reducing chronic neuroinflammation. Immunomodulatory therapies, including JAK-STAT pathway inhibitors and IL-15 blockade, are under investigation for their potential to mitigate inflammatory responses in the central nervous system. Additionally, neuroprotective agents such as minocycline and rapamycin are being explored for their ability to reduce oxidative stress and prevent neuronal damage. The efficacy of antiretroviral therapy (ART) in HTLV infection remains limited, as the virus persists predominantly in a clonal state rather than actively replicating. However, epigenetic agents such as azacytidine are being investigated for their ability to induce apoptosis in HTLV-infected cells and reverse viral latency mechanisms.

Despite being recently identified, HTLV-3 and HTLV-4 have not yet been associated with human diseases. However, ongoing epidemiological surveillance is crucial to determine their potential pathogenicity and clinical significance. Advances in next-generation sequencing (NGS) and phylogenetic analysis have improved our understanding of these novel HTLV strains, particularly in endemic regions of Central Africa [56,249].

Another major research priority is the development of a preventive HTLV vaccine [171,250,251,252,253], which remains an unmet need despite decades of investigation. Recent advancements in mRNA-based vaccine platforms and virus-like particles (VLPs) have reinvigorated vaccine research, with a focus on eliciting neutralizing antibody responses. Therapeutic vaccine strategies targeting HTLV-1 Tax and HBZ proteins are also being explored to enhance cell-mediated immunity in infected individuals.

Overall, the field of HTLV research and therapeutic development is advancing rapidly, with gene-editing, immunotherapy, and neuroprotective strategies offering new possibilities for disease management and eradication. While ATL and HAM/TSP remain significant clinical challenges, the continued exploration of immune-based interventions, targeted therapies, and viral latency disruption strategies holds substantial promise. The screening of HTLV-3 and HTLV-4 will be essential to assess their epidemiological relevance, and progress in vaccine development may ultimately provide a viable approach to preventing HTLV transmission in endemic populations. Future breakthroughs will require multidisciplinary collaborations across virology, oncology, and immunology to translate these advances into clinical practice and public health strategies.

## 9. HIV/HTLV Coinfection: Implications for Disease Progression and Management

Co-infection with HIV and HTLV, particularly HTLV-1 and HTLV-2, poses unique challenges to clinical management and understanding of disease progression, especially in endemic regions. HIV/HTLV-1 coinfection has consistently been associated with an accelerated progression to AIDS, higher immune activation, and reduced survival rates, whereas HIV/HTLV-2 coinfection appears to confer a relative immunological benefit or neutral effect on disease trajectory. HTLV-1 primarily infects CD4+ T lymphocytes, leading to polyclonal proliferation and persistent immune stimulation, a mechanism that significantly augments HIV replication and impairs immune regulation, even in the context of antiretroviral therapy (ART). This persistent immune activation is characterized by increased levels of IL-2, IFN-γ, and TNF-α, which not only drive HIV transcription but also contribute to a chronic inflammatory milieu, predisposing coinfected individuals to metabolic, cardiovascular, and neurocognitive complications, regardless of HIV viral load suppression or apparent CD4+ T cell counts [254]. In fact, HTLV-1-induced expansion of CD4+ cells can lead to misleadingly high CD4+ counts, masking underlying immune dysfunction and increasing the risk of opportunistic infections, including disseminated tuberculosis and cerebral toxoplasmosis, even at ostensibly protective CD4+ levels [254]. Moreover, coinfection with HTLV-1 has been linked to a diminished virologic and immunologic response to ART in some cohorts, further complicating therapeutic decisions and follow-up protocols [255]. For this reason, several experts recommend earlier initiation of ART in patients coinfected with HTLV-1, independent of traditional CD4+ thresholds, especially in regions with high HTLV-1 endemicity or in patients presenting with systemic inflammation or early clinical signs of disease progression.

Conversely, HTLV-2 exerts a contrasting biological influence on HIV progression. Unlike HTLV-1, HTLV-2 preferentially infects CD8+ T cells and seems to promote a more regulated immune response that mitigates the deleterious effects of chronic HIV infection. Several molecular mechanisms have been proposed to explain the protective immunomodulatory effects of HTLV-2: the upregulation of CCL3L1, a chemokine that competes with HIV for the CCR5 receptor and reduces viral entry into host cells; suppression of the JAK/STAT signaling pathway, thus limiting the cytokine-driven amplification of HIV; downregulation of T and NK cell activation markers; and modulation of host microRNA expression profiles that may indirectly alter HIV transcription and replication efficiency [256,257]. Notably, HIV/HTLV-2 coinfected individuals have demonstrated higher expression levels of cytotoxic enzymes such as granzyme A, granzyme B, and perforin in CD8+ lymphocytes, correlating with a lower HIV-1 proviral load and slower disease progression [258]. In longitudinal studies, patients with HTLV-2 coinfection had a lower incidence of AIDS-defining illnesses and a more stable immunologic profile during ART, supporting the notion of a protective or at least non-detrimental role of HTLV-2 in the HIV disease course [255]. While these findings do not justify a modification of ART regimens specifically for HTLV-2 coinfected patients, they do highlight the importance of considering HTLV status in the overall management plan, especially in terms of immunological monitoring and evaluation of comorbid risks.

Despite these differences, both HTLV-1 and HTLV-2 influence ART effectiveness through immune-mediated mechanisms. No definitive evidence exists suggesting that one antiretroviral regimen is superior to others in the context of coinfection; however, some authors have proposed that ART drugs with good central nervous system (CNS) penetration, such as zidovudine or abacavir, might offer additional benefits in cases of HTLV-1 coinfection complicated by HTLV-associated myelopathy (HAM) or other neuroinflammatory disorders, although clinical trials are lacking [103]. Moreover, the evaluation of therapy efficacy should extend beyond the traditional CD4+ count and viral load, incorporating inflammatory biomarkers, functional T-cell assays, and perhaps even HTLV proviral load quantification, to better stratify the risk of progression and tailor follow-up schedules. Interestingly, the reactivation of latent HTLV infection under ART or immune reconstitution inflammatory syndrome (IRIS) phenomena have also been described, although rarely, and remain an area requiring further study, particularly in coinfected populations from sub-Saharan Africa and South America, where diagnostic and monitoring tools for HTLV are limited.

As coinfection presents a diagnostic challenge, especially when asymptomatic, routine screening for HTLV in HIV-positive patients remains a debated issue, with some guidelines recommending it in endemic areas or in high-risk populations such as intravenous drug users and individuals from endemic countries. Early identification of coinfection is crucial not only for prognostic purposes but also to anticipate complications and evaluate potential ART failure in contexts where inflammatory markers remain elevated despite viral suppression. On a molecular level, the HTLV Tax proteins play a central role in viral replication and pathogenesis—Tax-1 (from HTLV-1) being more oncogenic and inflammatory than Tax-2 (from HTLV-2)—and their activity contributes to divergent cellular outcomes, from transformation to immune quiescence, further explaining the contrasting effects on HIV coinfection. This also opens potential therapeutic avenues, such as the development of Tax-targeted therapies or the use of anti-inflammatory agents to mitigate immune activation in HTLV-1 coinfection. The use of histone deacetylase inhibitors, JAK inhibitors, and other immune modulators has been explored in small studies and may eventually play a role in managing complex coinfections with persistent inflammation [255].

Overall, the interaction between HIV and HTLV—particularly HTLV-1—illustrates a paradigm in which coinfection does not merely represent the sum of two pathogens but a synergistic interplay that reshapes immune architecture, disease progression, and therapeutic responsiveness. While HTLV-2 coinfection may confer some immunologic benefits, HTLV-1 requires a more aggressive and nuanced clinical approach. Future studies should prioritize longitudinal, multi-center cohorts with integrated virologic, immunologic, and transcriptomic data to better define the optimal strategies for managing this dual infection, especially in under-resourced settings where both viruses continue to circulate and where coinfected patients remain at higher risk of rapid deterioration.

## 10. Conclusions

HTLVs continue to represent a significant yet often overlooked global public health challenge. Their oncogenic and neurotropic potential—especially that of HTLV-1—combined with their silent persistence in the majority of infected individuals, contributes to their clinical and epidemiological complexity. HTLV-1 is clearly associated with severe diseases such as ATL and HAM/TSP, while HTLV-2, although considered less pathogenic, remains prevalent among specific high-risk groups such as people who inject drugs and certain Indigenous populations. HTLV-3 and HTLV-4, found primarily in Central Africa, have not yet been definitively linked to human disease but warrant continuous surveillance due to their zoonotic origin.

The geographical distribution of HTLVs reveals marked differences among the virus types. HTLV-1 is endemic in areas like southwestern Japan, the Caribbean, South America, sub-Saharan Africa, Melanesia, and the Middle East. HTLV-2 shows a more restricted spread, particularly among Indigenous groups in the Americas and injecting drug users in non-endemic regions. These patterns are shaped by a complex interplay of cultural practices, migration, and transmission routes—including breastfeeding, sexual contact, and exposure to infected blood products.

While many prevalence estimates remain consistent over time, several regions have demonstrated notable temporal trends, particularly in response to targeted public health interventions. In Japan, for example, the implementation of nationwide HTLV-1 screening programs for blood donors and pregnant women, alongside changes in breastfeeding practices—such as advising seropositive mothers to refrain from breastfeeding—has led to a marked decline in prevalence among younger cohorts since the 1980s. These measures have proven effective in interrupting vertical transmission and limiting the spread of the virus across generations. Similarly, in Brazil and various Caribbean countries, the introduction of routine screening of blood donors, increased public awareness campaigns, and improvements in healthcare infrastructure have contributed to a reduction in HTLV-1 transmission through transfusions and possibly in the overall prevalence among the general population. However, these reductions are often unevenly distributed, with urban centers showing more pronounced declines compared to rural or underserved areas. Conversely, in sub-Saharan Africa, where HTLV-1 remains highly endemic, longitudinal data are scarce and surveillance systems are often fragmented. This lack of continuity in epidemiological monitoring hampers the ability to assess whether the prevalence is changing over time. In many of these settings, the absence of systematic screening, limited healthcare access, and cultural practices that may facilitate transmission contribute to the persistence of high infection rates. Without standardized, longitudinal data, it remains challenging to determine whether there are shifts in transmission dynamics or age-specific prevalence patterns in these populations. Understanding these temporal trends is crucial not only for evaluating the effectiveness of past interventions, but also for guiding future strategies aimed at HTLV prevention and control. Robust, ongoing surveillance systems, particularly in resource-limited and highly endemic regions, are essential to accurately assess the burden of disease and inform public health policies.

Despite decades of research, major gaps remain. In many regions, particularly in parts of Africa and Asia, the epidemiology of HTLV infections is still not well defined. Moreover, the lack of effective antiviral therapies and vaccines, alongside limited diagnostic resources in high-burden areas, hinders timely detection and clinical management. Socioeconomic disparities and healthcare access barriers further exacerbate disease outcomes and transmission dynamics.

Nonetheless, recent advances in molecular virology, host-pathogen interactions, and epigenetics are paving the way for promising therapeutic innovations—ranging from targeted epigenetic drugs and gene-editing strategies like CRISPR/Cas9 to personalized immunotherapies. Additionally, the integration of artificial intelligence and next-generation sequencing is enhancing our ability to identify high-risk carriers, detect early disease progression, and uncover new viral variants.

Addressing the burden of HTLVs requires a coordinated global effort. This includes strengthening surveillance systems, expanding culturally sensitive awareness campaigns, implementing broader screening programs, and developing region-specific risk reduction strategies. A multidisciplinary approach—combining scientific research, technological innovation, and equitable public health policies—is essential to reduce the clinical and social impact of these retroviruses and to ensure that affected populations are no longer neglected.

## Figures and Tables

**Table 1:** Summary of HTLV-1/2 endemic regions, prevalence, and data collection periods.

HTLV Type	Region	Key Countries/Areas	Prevalence Range (General Population/Specific Groups)	Data Collection Period
HTLV-1	Southwestern Japan	Kyushu, Okinawa	Blood donors: 1% (Hokkaido) to >6% (Kyushu, Okinawa)	2006–2016
	Sub-Saharan Africa	Gabon, DRC, Nigeria, Ghana, Guinea-Bissau	Adults: 0.3–3%; Older women (Gabon/DRC): 10–25%; Pregnant women (West Africa): 0.2–7.7%	early 2000s–2010s
	South America	Peru, Colombia, French Guiana, Brazil	Blood donors (Brazil): 0.04–1%	2000s–2010s
	Caribbean Area	Jamaica, Haiti	Jamaica (mean): 6.1%; Pregnant women (Haiti): 2.2–4.2%	1990s–2000s
	Middle East	Iran (Mashad region)	Adults: 0.77–3%	2003–2011
	Australo-Melanesia	Central Australia, PNG, Solomon Islands	Aboriginal Australians: up to 44%; Tribes: 1.2–3%	1990s–2000s
	Southeastern USA		Prevalence in blood donors, regional variations	2007–2015
HTLV-2	Indigenous populations of the Americas	Brazil (Amazon), Panama, USA	Kayapó: up to 41.2%; Native American tribes: up to 13%; Mexico: 0.23%	2000s–2010s
	People who inject drugs (PWID)	North America, Europe	Estimated prevalence: 20% (USA)	1990s–2010s
	Some Indigenous people in Africa	Cameroon, DRC (Pygmy populations)	Detected in Pygmy populations	2000s
	USA		Blood donors: HTLV-2 more common than HTLV-1; overall prevalence: 0.016%	2007–2015

**Table 2:** Summary of HTLV-1/2 transmission modes and populations at risk.

HTLV Type	Mode of Transmission	At-Risk Populations
HTLV-1	Mother-to-child transmission	Infants breastfed for prolonged periods by HTLV-1 positive mothers
	Sexual transmission	Sex partners of HTLV-1 infected individuals, particularly female partners of infected males
	Contaminated blood products	Recipients of unscreened blood transfusions or organ transplants
	Parenteral transmission	Intravenous drug users sharing needles
	Nosocomial transmission	Individuals undergoing medical procedures with inadequately sterilized equipment (suggested in Central Africa)
HTLV-1	Zoonotic transmission	Individuals with close contact to infected non-human primates, e.g., through bites (Central Africa)
	General Risk (Endemic Areas)	Residents of highly endemic regions, women (increased prevalence with age), specific ethnic groups (e.g., Aboriginal Australians)
HTLV-2	Mother-to-child transmission	Infants breastfed by HTLV-2 positive mothers
	Sexual transmission	Sex partners of HTLV-2 infected individuals
	Contaminated blood products	Recipients of unscreened blood transfusions or organ transplants
	Parenteral transmission	Intravenous drug users sharing needles
	General Risk (Endemic Areas)	Indigenous populations of the Americas, PWID, some Indigenous populations in Africa

**Table 3:** Overview of diagnostic methods for HTLV-1, HTLV-2, HTLV-3, and HTLV-4.

HTLV Type	Diagnostic Method	Test Type	Interpretation	Clinical Relevance
HTLV-1	Serology (Screening)	ELISA	Detects anti-HTLV-1/2 antibodies; requires confirmation due to cross-reactivity	First-line screening for HTLV infection
	Confirmatory Serology	Western Blot (WB)/Line Immunoassay (LIA)	Differentiates HTLV-1 from HTLV-2 based on specific viral protein bands	Confirms infection; may yield indeterminate results
	Molecular Testing	PCR	Detects HTLV-1 proviral DNA in PBMCs	Essential for confirming serology and diagnosing asymptomatic carriers
	Proviral Load Quantification	qPCR	Measures HTLV-1 proviral DNA levels	High proviral load associated with ATL and HAM/TSP
	Flow Cytometry	CCR4+ CD4+ T-cell analysis	Evaluates CCR4 expression in ATL cells	Used for prognosis and treatment decisions in ATL
HTLV-2	Serology (Screening)	ELISA	Detects HTLV-1/2 antibodies; requires differentiation from HTLV-1	Initial screening test
	Confirmatory Serology	Western Blot (WB)/Line Immunoassay (LIA)	Differentiates HTLV-2 from HTLV-1 by detecting specific viral proteins	Confirms HTLV-2 infection but may yield indeterminate results
	Molecular Testing	PCR	Detects HTLV-2 proviral DNA in PBMCs	Useful for confirmation in serologically indeterminate cases
	Proviral Load Quantification	qPCR	Measures HTLV-2 proviral DNA levels	HTLV-2 has lower pathogenicity; routine monitoring is not usually required
HTLV-3/HTLV-4	Serology	ELISA/Western Blot	Limited availability; assays still under development	Rarely tested in clinical settings due to uncertain pathogenicity
	Molecular Testing	PCR/Next-Generation Sequencing (NGS)	Identifies HTLV-3/HTLV-4-specific genetic sequences	Used for epidemiological research, not routine diagnosis

**Table 4:** Summary of HTLV types, associated diseases, diagnostic methods, and clinical manifestations.

HTLV Type	Associated Diseases	Diagnostic Methods	Main Symptoms and Clinical Features
HTLV-1	**1. Malignancies:** - ATL: Aggressive CD4+ T-cell malignancy with subtypes (acute, lymphoma, chronic, smoldering). **2. Neuroinflammatory Diseases:** - HAM/TSP: Chronic inflammatory demyelinating disorder affecting the spinal cord. **3. Inflammatory & Autoimmune Conditions:** - HTLV-1 associated uveitis (HAU): T-cell-mediated intraocular inflammation. - HTLV-1 associated polymyositis: Chronic inflammatory muscle disorder. - HTLV-1 associated arthritis: Immune-mediated joint inflammation. - HTLV-1 associated alveolitis: Interstitial lung disease due to lymphocytic infiltration. - Chronic infectious dermatitis (CID): Severe, recurrent skin infections, particularly in children.	**1. Serological testing:** - Enzyme-linked immunosorbent assay (ELISA): Detects anti-HTLV-1 antibodies. - Western Blot: Confirms seropositivity and distinguishes from HTLV-2. **2. Molecular testing:** - Polymerase Chain Reaction (PCR): Detects and quantifies HTLV-1 proviral DNA in peripheral blood mononuclear cells (PBMCs). - Southern Blot Analysis: Determines clonal integration in ATL. **3. Histopathology & Imaging:** - Bone Marrow Biopsy & Flow Cytometry (for ATL): Detects leukemic infiltration and abnormal CD4+/CD25+ T-cell expansion. - MRI of the Spinal Cord (for HAM/TSP): Shows atrophy, demyelination, and inflammatory changes in the thoracic spinal cord. - Slit-Lamp Biomicroscopy (for HAU): Reveals inflammatory cells in the anterior chamber and vitritis.	**1. ATL (Leukemia/Lymphoma Subtypes):** - Generalized lymphadenopathy, hepatosplenomegaly, skin lesions (erythematous plaques, nodules), hypercalcemia-induced nephropathy, lytic bone lesions, opportunistic infections. **2. HAM/TSP (Neurological Syndrome):** - Progressive lower limb spasticity and weakness, hyperreflexia, sensory deficits, urinary urgency/incontinence, erectile dysfunction, lumbar pain. **3. HTLV-1 Associated Uveitis:** - Blurred vision, floaters, photophobia, eye pain, granulomatous anterior uveitis, optic nerve involvement. **4. Polymyositis/Arthritis/Alveolitis:** - Muscle weakness, elevated creatine kinase (CK), interstitial lung fibrosis, chronic inflammatory arthritis with morning stiffness. **5. Chronic Infectious Dermatitis (CID):** - Persistent eczematous rash, secondary bacterial/fungal infections.
HTLV-2	**Possible but Unconfirmed Disease Associations:** - HTLV-2 is not definitively linked to specific malignancies or inflammatory disorders, but it has been implicated in neurological dysfunctions similar to HAM/TSP. - Some studies suggest increased susceptibility to opportunistic infections (e.g., bacterial pneumonia, urinary tract infections). - Possible link to neurodegenerative diseases, but conclusive evidence is lacking.	**1. Serological & molecular testing:** - ELISA & Western Blot: Differentiates HTLV-1 from HTLV-2. - PCR: Confirms proviral DNA integration of HTLV-2. **2. Neurological & Immune Function Assessments:** - Electromyography (EMG) & Nerve Conduction Studies (NCS): Detects subclinical neuropathies. - Cerebrospinal Fluid (CSF) Analysis: Occasionally shows pleocytosis and elevated IgG index.	**1. Neurological Symptoms (HAM/TSP-like Syndrome):** - Gait disturbances, muscle weakness, spasticity, bladder dysfunction, but with a slower and less aggressive progression than HTLV-1-associated HAM/TSP. **2. Immune Dysregulation & Infections:** - Recurrent bacterial and viral infections due to immune alterations. - Mild cognitive impairment reported in some cases.
HTLV-3	**No confirmed pathogenicity**: - HTLV-3 shares genetic similarities with HTLV-1, but no associated diseases have been identified. - Limited epidemiological data due to its recent discovery in Cameroon.	**Research-based diagnostic methods:** - PCR: Used for detecting HTLV-3 proviral DNA. - No commercially available serological tests.	**No known symptoms or clinical manifestations:** - Further research is needed to determine its pathogenic potential.
HTLV-4	**No known associated diseases:** - HTLV-4 was identified in a small number of asymptomatic individuals in Central Africa. - Its clinical significance is currently unknown.	Research-Based Diagnostic Methods: - PCR: Used for viral identification in experimental settings. - No available serological testing.	**No known symptoms or disease associations.**

**Table 5:** Overview of therapeutic strategies for HTLV-associated diseases.

Therapeutic Area	Strategy	Mechanism and Considerations
Gene-Editing Therapies	CRISPR-Cas9, ZFNs	Selective excision of HTLV-1 proviral DNA, prevention of oncogenic transformation. Challenges include delivery efficiency and off-target effects.
Immunotherapy	PD-1/PD-L1 inhibitors (nivolumab, pembrolizumab), CAR-T therapy	Enhances immune response against ATL but requires optimization to mitigate immune-related toxicity. CAR-T therapy is being developed to target CD4+ ATL cells.
Targeted Therapies for ATL	Mogamulizumab (anti-CCR4), lenalidomide, bortezomib	Depletes infected leukemic cells, modulates immune responses, and disrupts NF-κB-mediated survival pathways.
HAM/TSP Treatment	JAK-STAT inhibitors, IL-15 blockade, neuroprotective agents (minocycline, rapamycin)	Reduces neuroinflammation, slows disease progression, and protects against neuronal damage.
Antiviral Therapies	Azacytidine, conventional antiretroviral therapy	Limited efficacy of ART due to HTLV clonal persistence. Azacytidine shows promise in reactivating and eliminating infected cells.
HTLV-3 & HTLV-4 Surveillance	Epidemiological studies, genomic sequencing	Investigating potential disease associations and monitoring transmission dynamics in endemic regions.
Vaccine Development	mRNA-based vaccines, VLP-based vaccines, therapeutic vaccines (Tax/HBZ-based)	Aims to prevent HTLV-1 infection or enhance immune responses in infected individuals.

## Data Availability

The original contributions presented in the study are included in the article, further inquiries can be directed to the corresponding authors.
